# Indomethacin promotes survival of new neurons in the adult murine hippocampus accompanied by anti-inflammatory effects following MPTP-induced dopamine depletion

**DOI:** 10.1186/s12974-018-1179-4

**Published:** 2018-05-26

**Authors:** Elisabeth G. Hain, Maria Sparenberg, Justyna Rasińska, Charlotte Klein, Levent Akyüz, Barbara Steiner

**Affiliations:** 1 Charité – Universitätsmedizin Berlin, corporate member of Freie Universität Berlin, Humboldt – Universität zu Berlin and Berlin Institute of Health, Department of Neurology with Experimental Neurology, Charitéplatz 1, 10117 Berlin, Germany; 2 Charité – Universitätsmedizin Berlin, corporate member of Freie Universität Berlin, Humboldt – Universität zu Berlin, and Berlin Institute of Health, Institute for Medical Immunology, Augustenburger Platz 1, 13353 Berlin, Germany; 3 Charité – Universitätsmedizin Berlin, corporate member of Freie Universität Berlin, Humboldt – Universität zu Berlin, and Berlin Institute of Health, Berlin-Brandenburg Center for Regenerative Therapies (BCRT), Augustenburger Platz 1, 13353 Berlin, Germany

**Keywords:** Neurogenesis, Inflammation, Neurodegeneration, Parkinson’s disease, Indomethacin, MPTP, Hippocampus, Substantia nigra

## Abstract

**Background:**

Parkinson’s disease (PD) is characterized by dopaminergic cell loss and inflammation in the substantia nigra (SN) leading to motor deficits but also to hippocampus-associated non-motor symptoms such as spatial learning and memory deficits. The cognitive decline is correlated with impaired adult hippocampal neurogenesis resulting from dopamine deficit and inflammation, represented in the 1-methyl-4-phenyl-1,2,3,6-tetrahydropyridine hydrochloride (MPTP) mouse model of PD. In the inflammatory tissue, cyclooxygenase (COX) is upregulated leading to an ongoing inflammatory process such as prostaglandin-mediated increased cytokine levels. Therefore, inhibition of COX by indomethacin may prevent the inflammatory response and the impairment of adult hippocampal neurogenesis.

**Methods:**

Wildtype C57Bl/6 and transgenic Nestin-GFP mice were treated with MPTP followed by short-term or long-term indomethacin treatment. Then, aspects of inflammation and neurogenesis were evaluated by cell counts using immunofluorescence and immunohistochemical stainings in the SN and dentate gyrus (DG). Furthermore, hippocampal mRNA expression of neurogenesis-related genes of the Notch, Wnt, and sonic hedgehog signaling pathways and neurogenic factors were assessed, and protein levels of serum cytokines were measured.

**Results:**

Indomethacin restored the reduction of the survival rate of new mature neurons and reduced the amount of amoeboid CD68+ cells in the DG after MPTP treatment. Indomethacin downregulated genes of the Wnt and Notch signaling pathways and increased *neuroD6* expression. In the SN, indomethacin reduced the pro-inflammatory cellular response without reversing dopaminergic cell loss.

**Conclusion:**

Indomethacin has a pro-neurogenic and thereby restorative effect and an anti-inflammatory effect on the cellular level in the DG following MPTP treatment. Therefore, COX inhibitors such as indomethacin may represent a therapeutic option to restore adult neurogenesis in PD.

**Electronic supplementary material:**

The online version of this article (10.1186/s12974-018-1179-4) contains supplementary material, which is available to authorized users.

## Background

The hallmark of Parkinson’s disease (PD) is the dopaminergic cell loss in the substantia nigra (SN) leading to the characteristic motor deficits. Moreover, hippocampal non-motor functions such as spatial learning and memory are also impaired [1]. As nigral dopaminergic fibers project to the hippocampus, dopaminergic cell loss results in a deficit of the neurotransmitter dopamine in that brain area, affecting adult neurogenesis in the dentate gyrus (DG) of the hippocampus [2–5]. Animal models of PD have shown that this impaired neurogenesis following dopamine depletion correlates with PD-associated cognitive deficits [6–11]. These cognitive deficits together with a higher prevalence and earlier onset of PD are associated with the male gender [12, 13]. A commonly used animal model of the disease is the 1-methyl-4-phenyl-1,2,3,6-tetrahydropyridine hydrochloride (MPTP) mouse model. It represents the characteristic dopaminergic cell loss in the SN, determining the onset of PD [14, 15]. In this model, an impaired neurogenic process in the hippocampus and associated cognitive decline have also been reported [6–11]. In the SN, dopaminergic neurodegeneration leads to a release of soluble neuron-injury factors, which activate microglia [16, 17]. This results in the release of neurotoxic agents such as cytokines, reactive oxygen species, nitric oxide radicals, and prostaglandins (PG) and attracts lymphocytic infiltration of CD4+ and CD8+ T cells [17, 18]. All these events lead to a perpetuation of inflammation and neurodegeneration in the SN [17–21].

In the DG of the hippocampus of MPTP-treated mice, reactive microglia have also been observed [22, 23]. Microglial activation is known to alter the hippocampal microenvironment leading to a decreased survival of newly generated neurons in the DG [24–27]. The differentiation from newborn neural progenitor cells into functionally integrated neurons in the DG is a multistep process [28–30], which is highly vulnerable to pathological changes of the microenvironment such as inflammation [25, 27, 31]. Microglia-released pro-inflammatory cytokines such as interleukin (IL)-6 have been shown to be important factors between activated microglia and decreased hippocampal neurogenesis [27, 32]. In PD patients, pro-inflammatory cytokines are also elevated in the cerebrospinal fluid [33, 34], and microglial activation has been observed in the hippocampus of post-mortem brains [35, 36]. Thus, neurogenesis in the adult DG of PD patients might also be affected by inflammation in addition to the impaired homeostasis of the neurotransmitter dopamine.

The anti-inflammatory effect of the non-selective cyclooxygenase (COX) inhibitor indomethacin is based on a reduced PG production by inhibiting the basally expressed enzyme COX-1 and the inflammation-induced COX-2 [37, 38]. COX catalyzes the synthesis of PGH2 in a two-step process, which is accompanied by the production of neurotoxic free radicals [38, 39]. PGH2 is converted into biologically active PG such as PGE2, which leads to a rapid expression of COX-2 itself and other pro-inflammatory agents by activation of the PGE2 receptor 2 [38, 40]. A decreased inflammation by indomethacin treatment leads to the protection against neural cell loss in the hippocampus in animal models of cranial irradiation or ischemia [27, 41–45]. However, some studies described a decreased proliferation of hippocampal neuronal cells by COX inhibition in healthy and ischemic brains [46, 47]. Serrano and colleagues suggested a potential early neuroprotective role and a delayed neurodegeneration by COX-2 signaling [48].

Interestingly, COX-2 is upregulated in the SN of PD patients and in the MPTP mouse model [49] and leads to higher dopaminergic cell loss than in COX-2-deficient mice [50]. COX inhibition has been shown to prevent microglial activation and dopaminergic cell loss in the SN [51–53], which implies that COX plays a role in dopaminergic neurodegeneration.

This suggests that a PG-mediated inflammation represents a potential target. Thus, treatment with the non-selective COX inhibitor indomethacin might be a therapeutic option against dopaminergic cell loss and inflammation in the SN and the DG. We investigated here if indomethacin shows an anti-inflammatory effect on the cellular level, thereby reducing the levels of circulating pro-inflammatory and elevating the levels of anti-inflammatory cytokines, respectively. Furthermore, we studied the influence of indomethacin on different stages of adult hippocampal neurogenesis after MPTP treatment to test whether a therapy with indomethacin could be a suitable strategy to restore adult neurogenesis in PD patients.

## Methods

### Animals and housing

In total, 6- to 12-week-old female wildtype C57Bl/6N mice (*n* = 131; Charles River, Sulzfeld, Germany) and transgenic C57Bl/6N mice, expressing the green fluorescent protein (GFP) under the *nestin* promoter (Nestin-GFP) to label neural progenitor cells (*n* = 70, Forschungseinrichtungen für Experimentelle Medizin, Berlin, Germany) with a median weight of 21.1 g, were used. They were group-housed in standard cages in a temperature- and humidity-controlled standard colony room with a light-dark cycle of 12 h (starting at 6 am) and free access to food and water. Even though estradiol has a pro-neurogenic effect, adult hippocampal neurogenesis is not influenced by the sex itself in C57Bl/6 mice at the age investigated here [54–56].

All experiments were approved by the local animal ethics committee (Landesamt für Gesundheit und Soziales, Berlin, Germany) and carried out in accordance with the European Communities Council Directive of 22 September 2010 (10/63/EU).

### Group design and experimental procedure

**Fig. 1:**
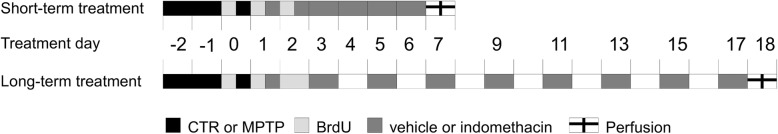
Timeline of the experimental procedure. MPTP: 1-methyl-4-(2′-methylphenyl)-1,2,3,6-tetrahydropyridine hydrochloride, BrdU: 5-bromo-2′-deoxyuridine

After 1 week of acclimatization, wildtype and Nestin-GFP mice were divided into two groups to receive either 2′-methyl-MPTP (further denoted as MPTP) or 0.9% saline as control (CTR) intraperitoneally (i.p.). Both groups were further assigned to short-term treatment (ST) groups and long-term treatment (LT) groups. Then, they were further divided to be treated either with indomethacin or 0.9% saline as vehicle i.p. over 6 consecutive days (ST) or every other day over 17 days (LT), starting after the last day of MPTP or saline injection. This results in eight groups with one control group (CTR + vehicle) in both time spans (ST and LT). All assignments into groups were performed pseudorandomly. After treatment cessation, mice were killed for histology and molecular analyses. A timeline of the experimental procedure is displayed in Fig. 1.

### MPTP mouse model

MPTP (generous gift from Prof. Dr. Christian Klein, Medicinal Chemistry, Institute of Pharmacy and Molecular Biotechnology IPMB, Heidelberg University, Heidelberg, Germany) was dissolved in 0.9% saline and injected i.p. at a dose of 20 mg/kg body weight in the morning hours on three consecutive days. CTR mice received three injections of saline instead. During MPTP injections, mice were treated in an extra room of the animal housing facility and transferred into an isolation cage from the first day of MPTP treatment until 2 days after due to the excrements containing MPTP and its metabolites.

### BrdU injections

5-Bromo-2′-deoxyuridine (BrdU, Sigma-Aldrich, Steinheim, Germany) was used for labeling proliferating cells. It was dissolved in 0.9% saline. All animals received BrdU i.p. at a dose of 50 mg/kg body weight in the morning hours on three consecutive days, starting at the last day of MPTP or saline, respectively.

### Indomethacin treatment

Indomethacin (Liometacen, Promedica Chiesi, Parma, Italy) was dissolved in distilled water and administered i.p. at a concentration of 2.5 mg/kg body weight in the morning hours either over 6 consecutive days (ST) or every other day over 17 days (LT), starting after the last day of MPTP or saline. Animals receiving 0.9% saline injections instead of indomethacin served as controls for the drug treatment (vehicle).

### Perfusion and tissue preparation

Animals were deeply anesthetized with ketamine/xylazine (10% Ketamine hydrochloride, WDT; 2% Rompun, Provet AG) i.p. before being transcardially perfused with phosphate-buffered saline (PBS) and 4% paraformaldehyde (PFA). The brains were removed from the skull, post-fixed in 4% PFA at 4 °C overnight, dehydrated with 30% sucrose solution at 4 °C for 48 h, and frozen at − 72 °C in 2-methylbutane (Sigma-Aldrich, Steinheim, Germany). Afterward, the brains were coronally sliced into 40-μm-thick sections using a Leica CM1850 UV cryostat and stored in cryoprotectant solution at 4 °C until histological analysis was performed.

For collecting blood samples and fresh brain tissue for molecular analysis, the animals were as well deeply anesthetized with ketamine/xylazine. Then, the abdomen was opened, and the blood samples were taken from the inferior vena cava. Aprotinin (Sigma-Aldrich, St. Louis, USA; 1 μl/1 ml blood sample) was added to the samples to prevent protein degradation. Samples were spun with an acceleration of 8000×*g* at 4 °C for 15 min, and sera were collected. After taking blood samples, the animals were transcardially perfused with PBS. Afterward, the brains were quickly removed from the skull and rapidly frozen on dry ice. The brains and serum samples were stored at − 80 °C until further analysis.

### Immunohistochemistry and cell quantification

For CD68 staining, antigen retrieval was performed on the brain sections using NaBH_3_. To continue with the immunohistochemical staining, a well-established protocol was followed [9]. One-in-six free-floating brain section series were treated with 0.6% H_2_O_2_. Hereafter, the sections for BrdU staining were also treated with 2 M HCl. After blocking with donkey serum-enriched PBS (PBS+), the sections were incubated overnight with the first antibody: anti-BrdU (rat, 1:500, AbD Serotec), anti-Iba-1 (rabbit, 1:1000, Wako), anti-CD68 (rat, 1:400, AbD Serotec), or anti-tyrosine hydroxylase (TH, mouse, 1:10,000, Sigma-Aldrich). The next day, the sections were incubated with the biotinylated secondary antibody (anti-rat, anti-rabbit, or anti-mouse, 1:250, dianova) at room temperature for 2 h. Afterward, an ABC solution to form a streptavidin-peroxidase complex (Vectastain ABC Elite Kit, Vector Laboratories) was applied, and the reaction was visualized by 3,3′-diaminobenzidine (DAB, Sigma-Aldrich)-nickel staining. Finally, the stained sections were mounted on microscope slides and coverslipped.

In total, the eight brain slices of the hippocampus (240 μm apart) of each mouse in the histological group were analyzed by manually counting BrdU-positive (BrdU+) cells in the subgranular zone and granular cell layer of the DG using the × 40 objective. Total numbers of Iba-1-positive (Iba+) cells and CD68-positive (CD68+) cells were counted manually in the eight brain slices of the wildtype mice in the hilus and granular and molecular layer of the DG using the × 40 objective. CD68+ cells were further subdivided into cells displaying an amoeboid or ramified shape. Amoeboid CD68+ cells are defined as cells with higher lysosomal activity, e.g., in microglia, macrophages, and to a lesser extent in dendritic cells, indicating a phagocytotic state [57]. Here, CD68+ cells were identified as amoeboid, if cell somas appear more round-shaped and more color-intense with no or only a few branches [58, 59]. In contrast, ramified CD68+ cells are characterized by a small cell body with thin processes [58, 59]. Numbers of amoeboid CD68+ cells were assessed by manual counting using the × 40 objective. Numbers of ramified CD68+ cells were estimated by taking the difference between all CD68+ cells and amoeboid CD68+ cells. For manual cell counting in the SN, including pars compacta and pars reticulata, four stained brain slices (240 μm apart) in total were analyzed for amoeboid CD68+ cells in the SN of wildtype mice and TH-positive (TH+) cells of Nestin-GFP mice using the × 40 objective. All manually assessed cell counts were done using an Axioskop HB50/AC light microscope (Zeiss, Germany) and multiplied by six to estimate the absolute cell numbers. A Stereo Investigator (MBF Bioscience) and a Leica DMRE microscope were used for quantification of the total numbers of Iba-1+ cells and CD68+ cells in the SN of wildtype mice. The region of interest was tracked with a × 5 and × 4 objective, respectively. Actual counting was done with a × 40 oil and × 20 objective, respectively, on four sections with a sampling grid size of 150 × 120 μm and a counting frame of 60 × 60 μm without guard dissector height. Cells were counted when cells bodies became sharp in their widest extent. The total amount of Iba-1+ and CD68+ cells was automatically estimated using the counted cell number, sampling grid size, counting frame size, slice interval, and slice thickness. The coefficient of error (Gundersen, *m* = 1) was ≤ 0.9. The numbers of ramified CD68+ cells in the SN were estimated by taking the difference between all CD68+ cells and amoeboid CD68+ cells. All data were collected blinded to the treatment groups.

### Immunofluorescence and cell quantification

For the characterization of newly generated BrdU+ cells in the DG following the stages of neuronal development in the adult DG [29] (Additional file 1: Figure S1), the brain slices were triple-stained for BrdU, Nestin, visualized by co-expressed GFP (Nestin-GFP), and doublecortin (DCX) in Nestin-GFP mice or BrdU, DCX, and neuronal nuclei (NeuN) in wildtype mice, following a well-established protocol [9]. Briefly, one-in-twelve free-floating brain sections (480 μm apart) were pre-treated with 2 M HCl and blocked with PBS+. Sections were then incubated with anti-BrdU (rat, 1:500, AbD Serotec), anti-GFP (rabbit, 1:200, Abcam), anti-DCX (goat, 1:100, Santa Cruz Biotechnology), and anti-NeuN (mouse, 1:1000, Abcam) at 4 °C overnight. The next day, the sections were incubated with fluorescent secondary antibodies RhodamineX (anti-rat, 1:250, dianova), Alexa488 (anti-rabbit or anti-mouse, 1:1000, invitrogen), and Alexa647 (anti-goat, 1:300, dianova) at room temperature for 4 h, mounted on microscope slides and coverslipped.

To evaluate the number of newly generated cells following the stages of neurogenesis (Additional file 1: Figure S1), 50 BrdU+ cells within the subgranular zone and the granule cell layer were detected using a confocal microscope (TCS SP2, Leica, Wetzlar, Germany) under a × 63 objective and were analyzed for co-labeling with Nestin-GFP-positive (BrdU+/Nestin-GFP+) type 1 cells, triangular shaped cells with an apical process, and type 2a cells with short, tangentially orientated processes, Nestin-GFP-positive/DCX-positive (BrdU+/Nestin-GFP+/DCX+) type 2b cells, DCX-positive type 3 cells (BrdU+/DCX+) including immature neurons or NeuN-positive (BrdU+/NeuN+) mature neurons. Hereof, the absolute numbers were estimated by the ratio of co-labeled BrdU+ cells to all BrdU+ cells. All data were collected blinded to the treatment groups.

### Measurement of cytokine concentration

To assess peripheral inflammatory processes following MPTP treatment, the protein levels of six representative cytokines were measured in the serum: interleukin (IL)-1β, IL-6, IL-10, IL-17a, interferon (IFN)-γ, and tumor necrosis factor (TNF)-α. For the detection, a Bio-Plex Pro™ Mouse Cytokine Th17 Panel A 6-Plex Group 1 kit (Bio-Rad Laboratories, Inc.) and a Bio-Plex^®^ 200 System (Bio-Rad Laboratories, Inc.) plate reader were used. Serum samples were applied undiluted. The assay was processed following the manufacturer’s protocol (Bio-Plex Pro TM Cytokine, Chemokine, and Growth Factor Assays Instruction Manual, Bio-Rad Laboratories, Inc.).

### mRNA isolation and gene expression analysis

To investigate possible altered signaling pathways in neurogenesis, the expression of *gli1*, *hes5*, *lef1*, effector genes of the sonic hedgehog, Notch and Wnt signaling pathways, respectively, and of the pro-neurogenic factors *neuroD6* and *ngn1* in the hippocampus was assessed. Therefore, the samples (1 mm in diameter) were taken from the brain slices of the anterior hippocampus (Bregma − 1.82 to − 2.3 mm). Hippocampal total RNA was isolated with the Nucleospin RNA/Protein isolation kit (Macherey-Nagel, Düren, Germany) and reverse transcribed using the High Capacity RNA-to-cDNA kit (Applied Biosystems, CA, USA). cDNA corresponding to 1 ng of total RNA was used for gene expression analysis carried out with the StepOne real-time PCR instrument and software (Applied Biosystems, CA, USA). The amplification was performed with TaqMan assays (*gapdh*: Mm99999915_g1, *gli1*: Mm00494654_m1, *hes5*: Mm00439311_g1, *lef1*: Mm00550265_m1, *neuroD6*: Mm01326464_m1, *ngn1*: Mm00440466_s1) according to the TaqMan Fast Advanced Master Mix protocol (Applied Biosystems, CA, USA). Relative gene expression was calculated with the comparative C_t_ method (ΔΔC_t_) and *gapdh* as the reference gene. Data are displayed as fold change compared to CTR + vehicle.

### Statistical analysis

Data of the ST and LT groups were analyzed separately by using IBM SPSS Statistics 25 for Windows and GraphPad Prism 7. A 2 × 2 factorial design with the between-subject factors neurotoxin (CTR vs. MPTP) and drug (vehicle vs. indomethacin) was used. The two-way between-subjects ANOVA was performed for histological, Multiplex ELISA, and real-time PCR data to test the main effects of the factors neurotoxin and drug and their interaction. Pairwise comparison using the Bonferroni test was done in case of a significant interaction. *P* values ≤ 0.05 were considered statistically significant. All histological data and real-time PCR data are displayed in box plots with a center line as median and whiskers indicating the minimum and maximum value. Multiplex ELISA data are given tabularly as mean ± SEM. Graphs were created using GraphPad Prism 7.

## Results

### Indomethacin prevents the MPTP-induced decrease in the number of new mature neurons in the DG

**Fig. 2:**
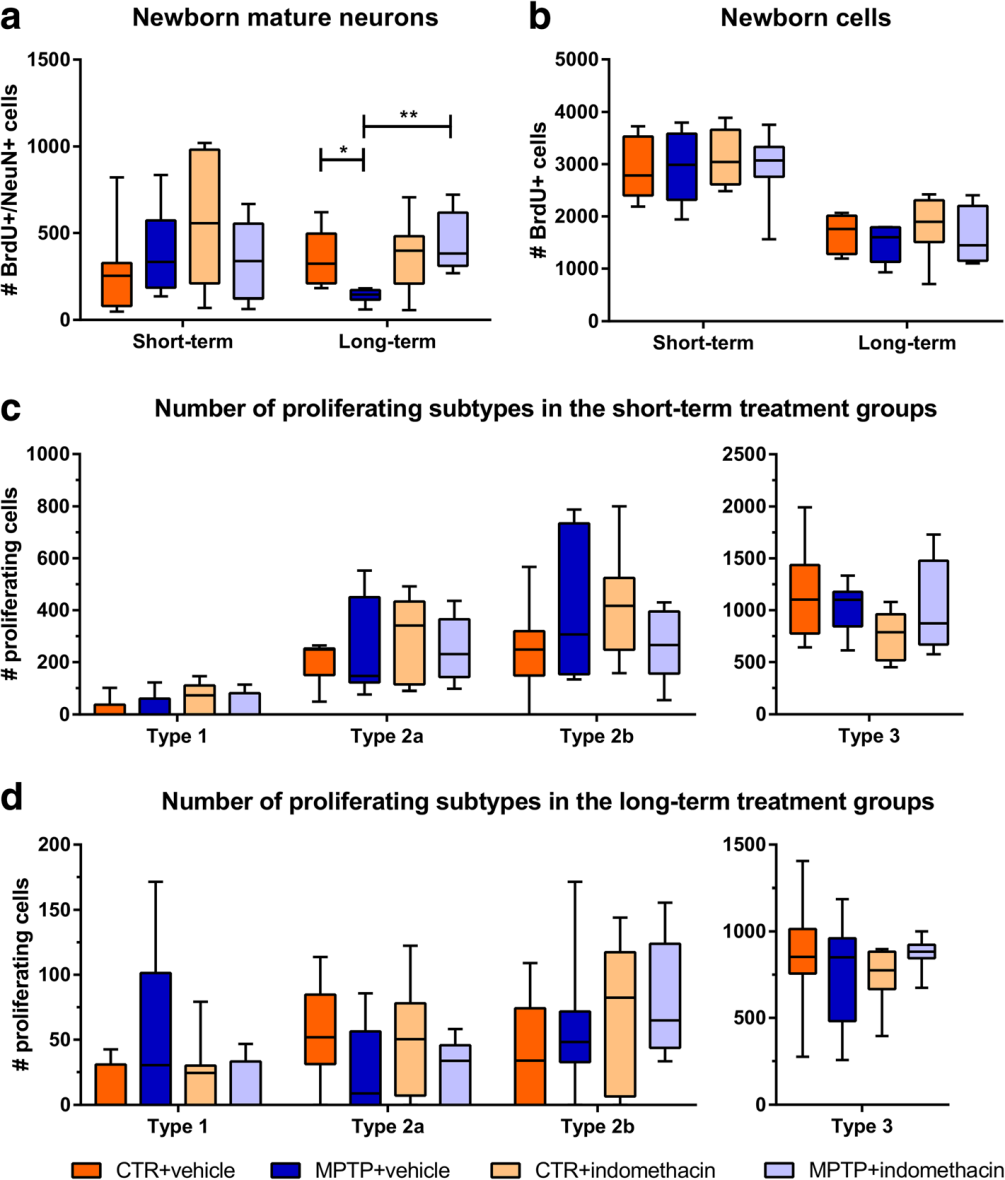
Results of histological cell counts of proliferating cells in the dentate gyrus. Absolute numbers of newborn mature neurons (**a**) and all newborn cells (**b**), and subtypes of newborn progenitor cells in short-term- (**c**) and long-term-treated (**d**) mice in the dentate gyrus revealed by immunohistological and immunofluorescent analysis. *N* = 6–8/group. A two-way ANOVA with factors neurotoxin, drug, and their interaction was performed. A significant interaction was followed by a Bonferroni post hoc test with *p ≤ 0.05, **p ≤ 0.01. CTR: control; MPTP: 1-methyl-4-(2′-methylphenyl)-1,2,3,6-tetrahydropyridine hydrochloride

In the total cell count of BrdU+/NeuN+ mature granule cells in the LT group, a significant interaction (*F*(1,26) = 5.413) and a significant main effect of the factor drug (*F*(1,26) = 7.658) were observed. Pairwise comparison showed that MPTP treatment reduced the number of new mature neurons compared to CTR (CTR + vehicle vs. MPTP + vehicle, *p* ≤ 0.05). Indomethacin treatment prevented this reduction (MPTP + vehicle vs. MPTP + indomethacin, *p* ≤ 0.01) (Fig. 2a, Additional file 1: Figure S1). In CTR mice, the number of newly generated neurons was not altered by indomethacin (CTR + vehicle vs. CTR + indomethacin, *p* ≥ 0.05).

### Total number of proliferating cells is not affected by MPTP or indomethacin

At both time points, no significant alterations by the factors neurotoxin (ST: *F*(1,28) = 0.086; LT: *F*(1,26) = 1.422), drug (ST: *F*(1,28) = 0.382; LT: *F*(1,26) = 0.511), and their interaction (ST: *F*(1,28) = 0.222; LT: *F*(1,26) = 0.005) in the total number of BrdU+ cells were observed (Fig. 2b).

### Neurotoxin treatment decreases the number of proliferating type 2a cells in the DG

**Fig. 3:**
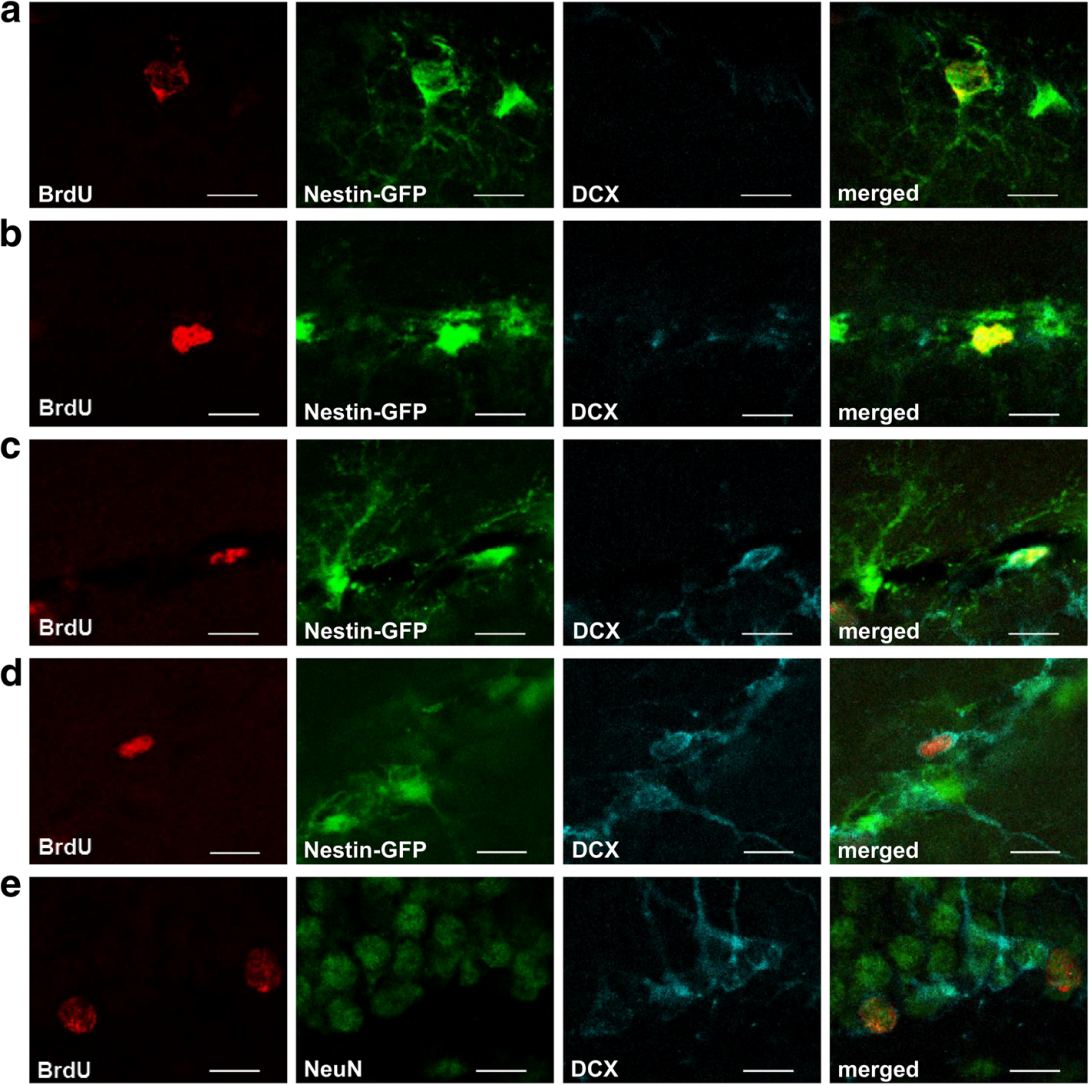
Representative confocal images of the subtypes. Proliferating subtypes of neurogenesis in the dentate gyrus: progenitor cell type 1 (**a**), progenitor cell type 2a (**b**), progenitor cell type 2b (**c**), progenitor cell type 3 (**d**), and mature neuron (**e**). Scale bars indicate 10 μm. BrdU: 5-bromo-2′-deoxyuridine; DCX: doublecortin; GFP: green fluorescent protein; NeuN: neuronal nuclei

A significant interaction of the factors neurotoxin and drug was detected in the number of BrdU+/Nestin-GFP+/DCX+ type 2b cells in the ST group (*F*(1,26) = 4.325) (Fig. 2c), but no relevant significant difference in the post hoc Bonferroni test. A significant main effect of the factor neurotoxin in the total number of newly generated BrdU+/Nestin-GFP+ type 2a cells was revealed in the LT group (*F*(1,28) = 4.893) (Fig. 2d). There were no effects of the factors neurotoxin and drug alone or in their interaction on the absolute numbers of BrdU+/Nestin-GFP+ type 1 cells, BrdU+/Nestin-GFP+ type 2a cells in the ST group and BrdU+/Nestin-GFP+/DCX+ type 2b cells in the LT group as well as BrdU+/DCX+ type 3 cells including immature neurons at both time points (Fig. 2c, d). Representative images of the different cells types are shown in Fig. 3a–e.

### Indomethacin and MPTP transiently downregulate Wnt signaling, whereas drug treatment alone upregulates *neuroD6* expression in the hippocampus

**Fig. 4:**
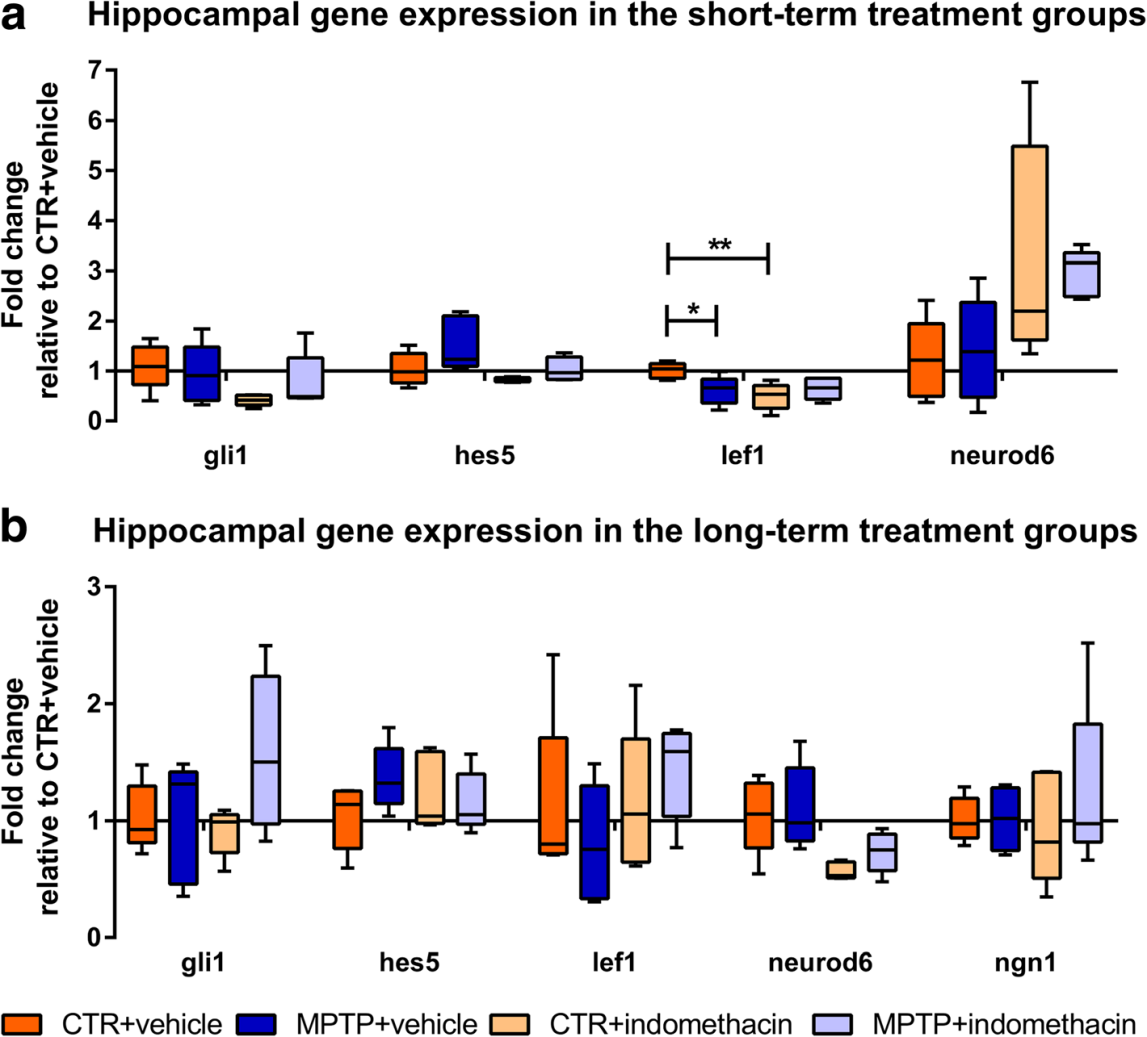
Hippocampal gene expression analysis. Quantitative real-time PCR was performed for the effector genes *gli1*, *hes5*, and *lef1* of the sonic hedgehog, Notch, and Wnt signaling pathways, respectively, and for the neurogenic factors *neuroD6* and *ngn1* in short-term- (**a**) and long-term-treated (**b**) mice. Gene expression is displayed as fold changes of mRNA levels in relation to CTR + vehicle. *N* = 5/group. A two-way ANOVA with factors neurotoxin, drug, and their interaction was performed. A significant interaction was followed by a Bonferroni post hoc test: *p ≤ 0.05, **p ≤ 0.01. CTR: control; MPTP: 1-methyl-4-(2′-methylphenyl)-1,2,3,6-tetrahydropyridine hydrochloride

In the ST group, there was a significant interaction (*F*(1,16) = 7.067) and a main effect of the factor drug (*F*(1,16) = 5.275) in the mRNA expression of *lef1*, an effector of the Wnt signaling pathway. Here, the pairwise comparison showed a reduced *lef1* expression by MPTP and indomethacin treatment compared to CTR mice (CTR + vehicle vs. MPTP + vehicle, *p* ≤ 0.05; CTR + vehicle vs. CTR + indomethacin, *p* ≤ 0.01) (Fig. 4a). These effects were no longer present in the LT group. The factor drug increased mRNA expression of the pro-neurogenic basic helix-loop-helix (bHLH) gene *neuroD6* in the ST group (*F*(1,16) = 9.530) and in the LT group (*F*(1,16) = 13.548). Significant main effects of the factor neurotoxin (*F*(1,16) = 5.469) and factor drug (*F*(1,16) = 5.482) on mRNA expression of the anti-neurogenic bHLH repressor gene *hes5*, an effector of the Notch signaling pathway, were revealed in the ST group. These effects were no longer present in the LT group. No mRNA expression of the neurogenic factor *ngn1* could be measured in the ST group. No significant effects of the factors neurotoxin, drug, and their interaction were revealed on the mRNA expression of *ngn1* in the LT group and of *gli1*, an effector gene of the sonic hedgehog signaling pathway, at both time points. Data of mRNA levels are presented in Fig. 4 and in the supplementary material (Additional file 2: Table S1).

### Reduced numbers of amoeboid CD68+ cells in the DG of MPTP mice following indomethacin treatment

**Fig. 5:**
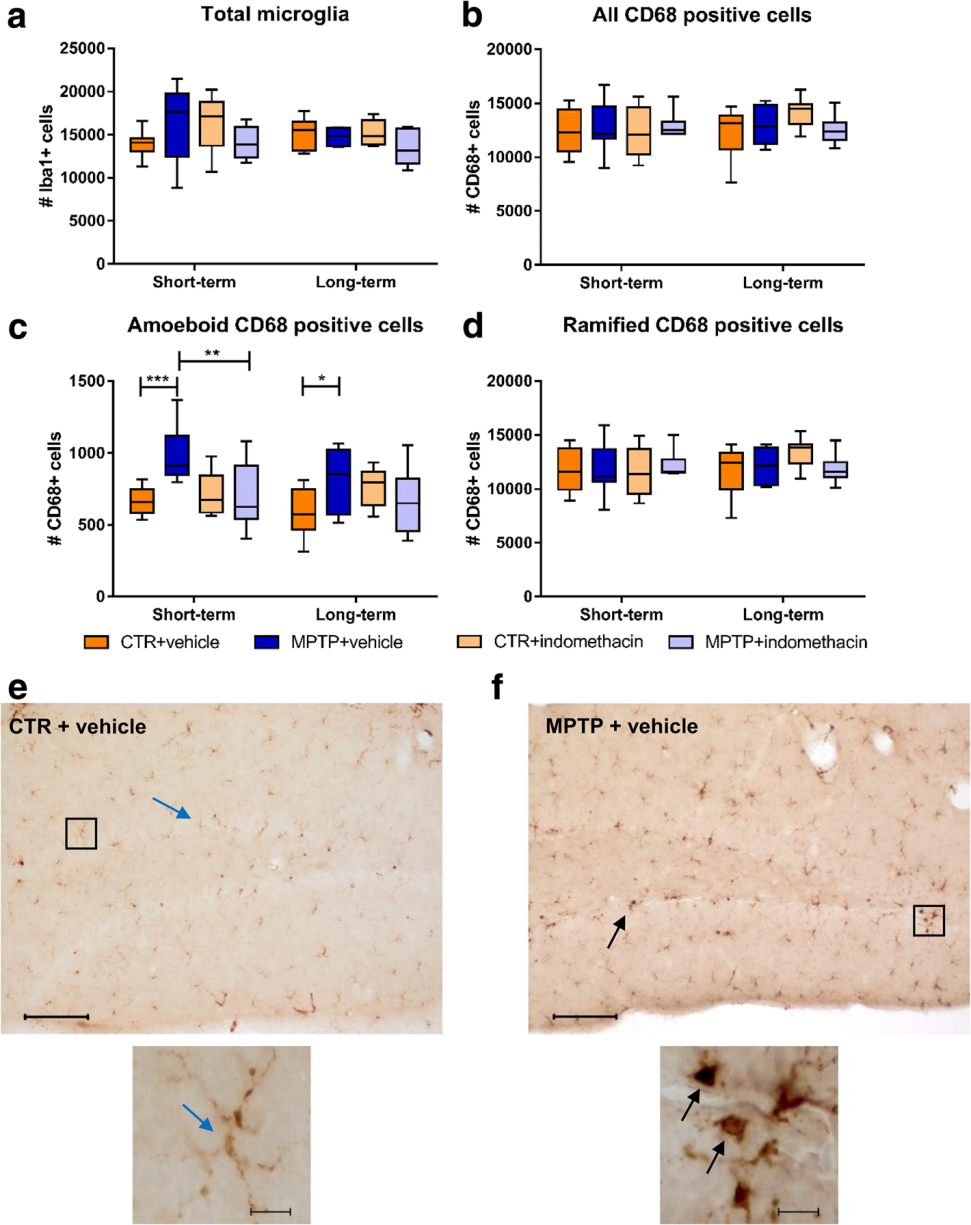
Results of histological cell counts of cellular inflammation in the dentate gyrus. Absolute numbers of Iba-1-positive cells (**a**), CD68-positive cells (**b**), amoeboid CD68-positive cells (**c**), and ramified CD68-positive cells (**d**) and representative images of CD68-positive cells in CTR mice (**e**) and MPTP-treated mice (**f**). Black arrows indicate amoeboid CD68-positive cells, and blue arrows indicate ramified CD68-positive cells. In the short-term treatment group, indomethacin reduces the increased number of amoeboid CD68-positive cells after dopamine depletion. Scale bars indicate 100 and 10 μm in the higher magnification. *N* = 6–8/group. A two-way ANOVA with main factors neurotoxin, drug, and their interaction was performed. A significant interaction was followed by Bonferroni post hoc test with *p ≤ 0.05, **p ≤ 0.01, ***p ≤ 0.001. CTR: control; MPTP: 1-methyl-4-(2′-methylphenyl)-1,2,3,6-tetrahydropyridine hydrochloride

There was a significant interaction of neurotoxin and drug in the total count of Iba-1+ microglia in the ST group (*F*(1,28) = 4.497). Post hoc analysis showed no significant differences (Fig. 5a). No significant main effects of the factors neurotoxin and drug or significant interaction on the total numbers of CD68+ cells were found (Fig. 5b). At both time points, the interaction of neurotoxin and drug led to significantly different amounts of amoeboid CD68+ cells (ST: *F*(1,28) = 7.923; LT: *F*(1,26) = 5.860). The pairwise comparison revealed a significantly higher cell count of amoeboid CD68+ cells in MPTP-treated mice compared to CTR mice at both time points (CTR + vehicle vs. MPTP + vehicle: ST *p* ≤ 0.001; LT *p* ≤ 0.05). Indomethacin prevented this in the ST group (MPTP + vehicle vs. MPTP + indomethacin, *p* ≤ 0.01) (Fig. 5c). There were also significant main effects of the factor neurotoxin (*F*(1,28) = 5.767) and drug (*F*(1,28) = 4.298) on the amount of amoeboid CD68+ cells in the ST group. No significant main effects or interaction on the numbers of ramified CD68+ cells could be detected at both time points (Fig. 5d). Representative images of CD68+ cells are shown in Fig. 5e, f.

### Long-term indomethacin treatment prevents MPTP-induced increase of IL-10 and IL-17a levels in serum

A significant interactive effect of the factors neurotoxin and drug was detected in the serum levels of IL-10 (*F*(1,31) = 4.432) and IL-17a (*F*(1,29) = 7.825) in the LT group. The pairwise comparison revealed a significantly higher serum concentration of the anti-inflammatory cytokine IL-10 and the pro-inflammatory cytokine IL-17a in MPTP-treated mice compared to CTR mice (CTR + vehicle vs. MPTP + vehicle, *p* ≤ 0.05 and *p* ≤ 0.01, respectively). Treatment with indomethacin following MPTP prevented this increase (MPTP + vehicle vs. MPTP + indomethacin, *p* ≤ 0.01). In the ST group, no significant effect of either the factors neurotoxin, drug, or their interaction was found. No significant change was revealed for the serum concentrations of the pro-inflammatory cytokines IL-1β, IL-6, IFN-γ, and TNF-α at either time point (Table 1).

**Table 1:** Serum protein levels of cytokines

		Cytokine					
		IL-1β	IL-6	TNF-α	IL-17a	IFN-γ	IL-10
Short-term treatment	CTR + vehicle	123.7 ± 65.7	9.9 ± 1.8	481.2 ± 93.0	22.0 ± 4.8	26.4 ± 4.7	61.4 ± 11.2
	MPTP + vehicle	180.8 ± 36.5	11.7 ± 2.2	500.8 ± 104.2	39.0 ± 17.7	27.3 ± 5.3	68.6 ± 13.3
	CTR + indomethacin	255.2 ± 30.2	11.6 ± 2.0	537.2 ± 91.4	27.1 ± 4.4	29.9 ± 5.1	76.4 ± 12.1
	MPTP + indomethacin	157.5 ± 76.5	9.1 ± 1.3	442.8 ± 91.5	22.8 ± 2.1	26.2 ± 1.8	70.4 ± 4.3
Long-term treatment	CTR + vehicle	94.0 ± 36.0	8.6 ± 1.1	406.3 ± 53.7	15.5 ± 2.2	23.9 ± 3.0	54.7 ± 7.1
	MPTP + vehicle	151.4 ± 29.6	11.5 ± 1.9	569.5 ± 82.3	38.2 ± 6.4**	30.0 ± 3.9	88.6 ± 9.7*
	CTR + indomethacin	92.0 ± 35.1	10.7 ± 1.7	466.8 ± 89.9	24.6 ± 4.9	27.0 ± 4.3	60.1 ± 10.2
	MPTP + indomethacin	126.9 ± 26.7	9.4 ± 1.3	354.8 ± 73.6	19.3 ± 3.6^##^	24.8 ± 4.2	52.5 ± 9.9^##^

Multiplex ELISA was performed to evaluate the levels of pro-inflammatory IL-1β, IL-6, TNF-α, IL-17a, and IFN-γ and anti-inflammatory IL-10 in serum of short-term and long-term groups. Values are expressed as mean ± SEM in pg/ml, *n* = 7–11/group. A two-way ANOVA with factors neurotoxin, drug, and their interaction was performed

*CTR* control, *MPTP* 1-methyl-4-(2′-methylphenyl)-1,2,3,6-tetrahydropyridine hydrochloride

A significant interaction was followed by Bonferroni post hoc test: *p ≤ 0.05, **p ≤ 0.01 compared to CTR + vehicle; ^##^*p* ≤ 0.01 compared to MPTP + vehicle

### Indomethacin prevents MPTP-induced increase of Iba-1+ cell numbers, and drug treatment alone decreases the amount of CD68+ amoeboid cells in the SN

**Fig. 6:**
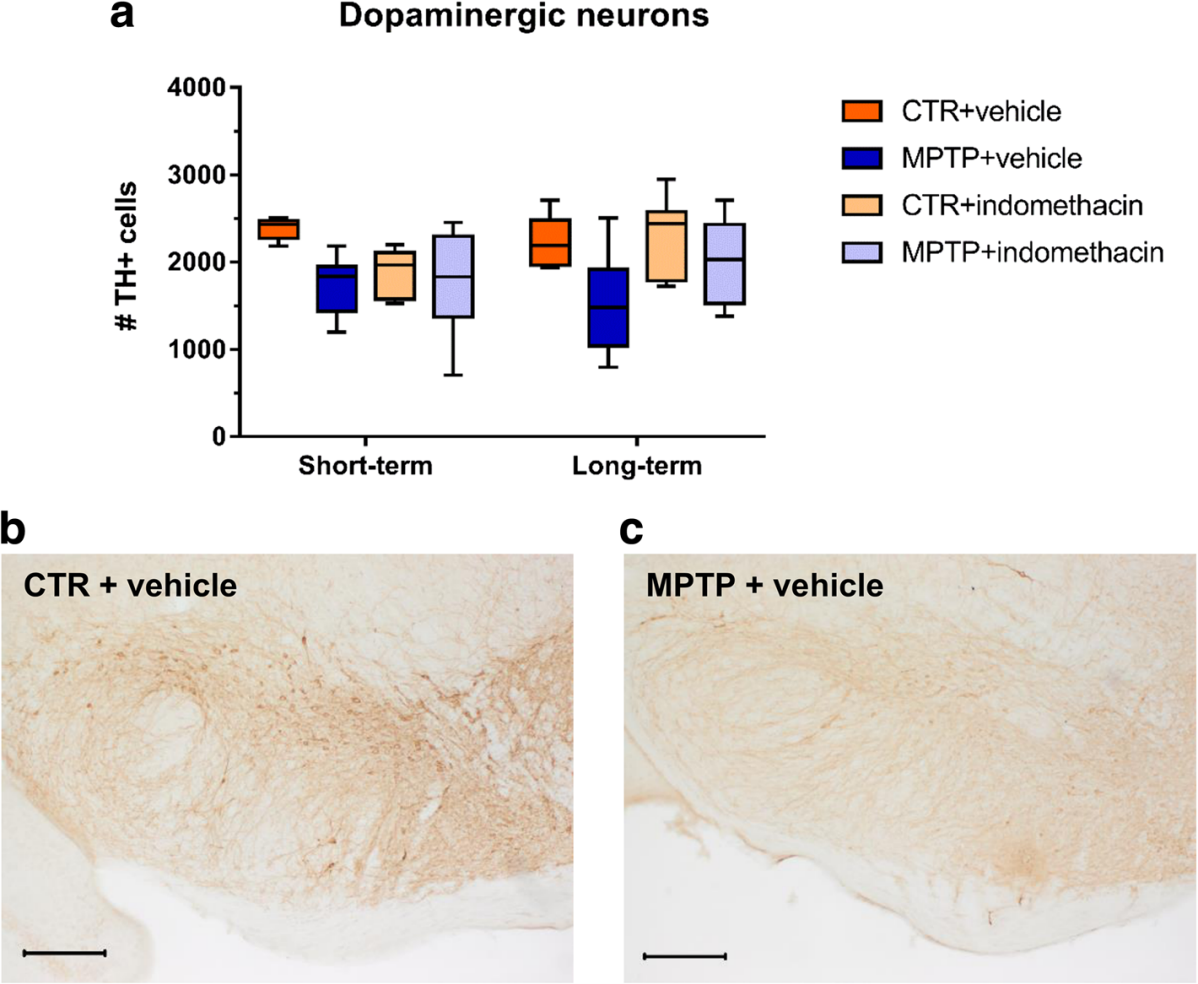
Results of histological cell counts and representative images of dopaminergic neurons in the substantia nigra. Absolute numbers of TH-positive cells (**a**) and representative images, displaying the dopaminergic cell loss after neurotoxic treatment in the substantia nigra in mice of the CTR + vehicle (**b**) and MPTP + vehicle (**c**) group, representatively. Scale bars indicate 200 μm. *N* = 6–11/group. CTR: control; MPTP: 1-methyl-4-(2′-methylphenyl)-1,2,3,6-tetrahydropyridine hydrochloride

A two-way ANOVA revealed a significant main effect of the factor neurotoxin on the number of TH+ neurons at both time points (ST: *F*(1,26) = 8.609; LT: *F*(1,33) = 11.303), but no effect of drug (ST: *F*(1,26) = 2.573; LT: *F*(1;33) = 3.189) or interaction (ST: *F*(1,26) = 3.821; LT: *F*(1,33) = 2.082) (Fig. 6a). As dopaminergic cell loss by neurotoxic treatment was achieved, the here selected time points in this model represent the onset of PD. Representative images, displaying the reduction of TH+ cells after neurotoxic treatment, are represented in Fig. 6b, c.

**Fig. 7:**
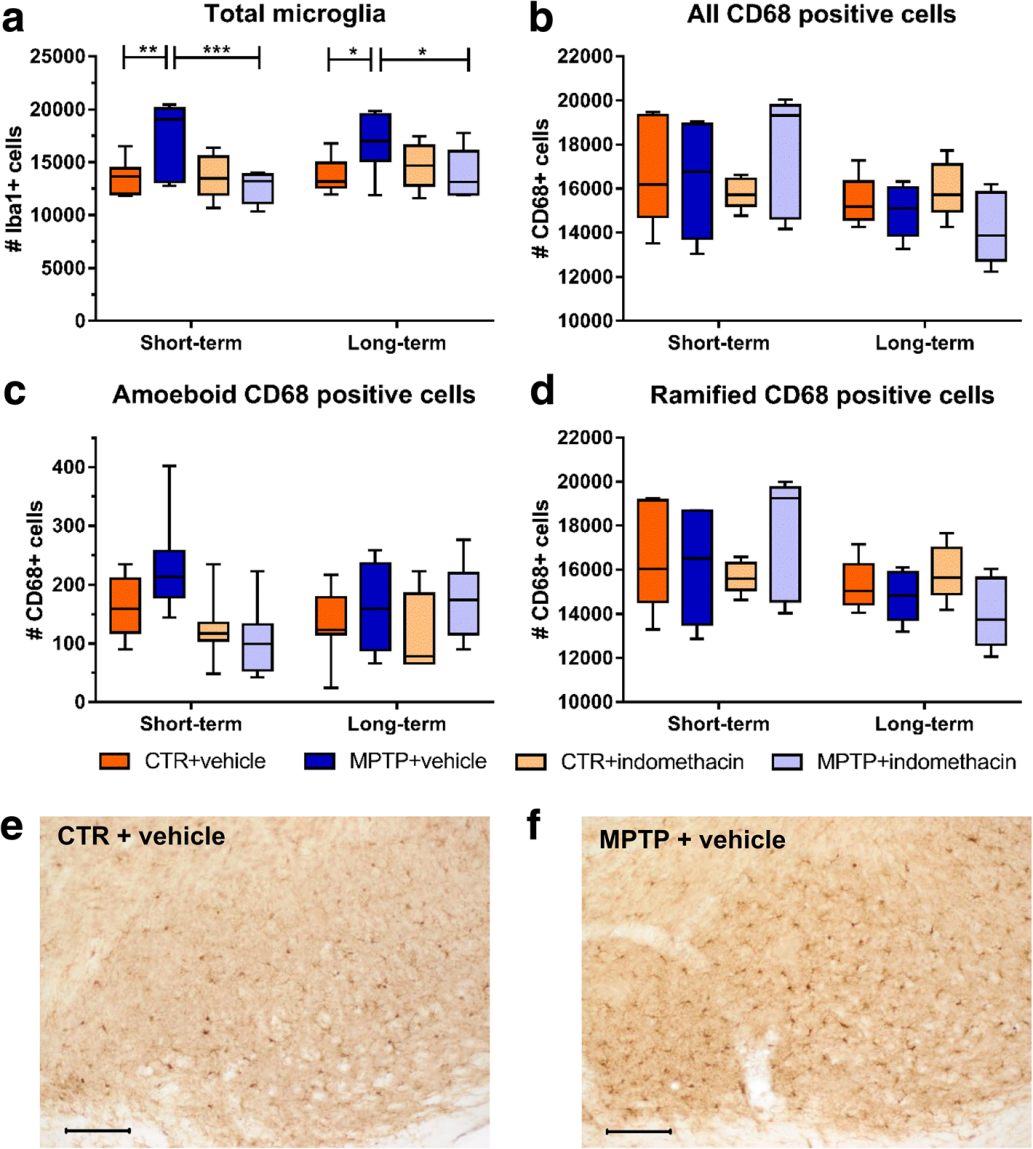
Results of histological cell counts and representative images of cellular inflammation in the substantia nigra. Absolute numbers of Iba-1-positive cells (**a**), CD68-positive cells (**b**), amoeboid CD68-positive cells (**c**), and ramified CD68-positive cells (**d**) and representative images of Iba-1-positive cells in CTR mice (**e**) and MPTP-treated mice (**f**), displaying the higher amount of Iba-1-positive cells after dopamine depletion in the substantia nigra. Scale bars indicate 100 μm. *N* = 5–8/group. A two-way ANOVA with main factors neurotoxin, drug, and their interaction was performed. A significant interaction was followed by Bonferroni post hoc test with *p ≤ 0.05, **p ≤ 0.01, ***p  ≤ 0.001. CTR: control; MPTP: 1-methyl-4-(2′-methylphenyl)-1,2,3,6-tetrahydropyridine hydrochloride

A significant interaction of both factors in the amount of Iba-1+ cells was observed in the ST group (*F*(1,28) = 7.715) and LT group (*F*(1,26) = 5.800). At both time points, the pairwise comparison showed significantly higher numbers of Iba-1+ microglia in MPTP-treated mice than in CTR mice (CTR + vehicle vs. MPTP + vehicle: ST, *p* ≤ 0.01; LT, *p* ≤ 0.05). This was decreased following indomethacin treatment (MPTP + vehicle vs. MPTP + indomethacin: ST, *p* ≤ 0.001; LT, *p* ≤ 0.05) (Fig. 7a). A significant main effect of the factor drug was revealed in the amount of Iba-1+ cells in the ST group (*F*(1,28) = 8.293). On the numbers of all CD68+ cells, no significant effect of either the factors neurotoxin, drug, or their interaction was found (Fig. 7b). In the ST group, a significant main effect of the factor drug could be observed on the amount of amoeboid CD68+ cells (*F*(1,28) = 14.753) (Fig. 7c). No significant main effects or interaction of both factors on the number of ramified CD68+ cells was observed at both time points (Fig. 7d). Representative images of Iba-1+ cells are presented in Fig. 7e, f.

## Discussion

We here demonstrate that indomethacin is effective in preventing the impaired neurogenic process in the adult hippocampus following MPTP-induced dopamine depletion and has an anti-inflammatory effect by reducing the number of amoeboid CD68+ cells as one factor of inflammation. Both MPTP treatment and inflammation are known to decrease the survival of newly generated neurons in the DG [7, 9, 27, 60]. As MPTP treatment itself is also accompanied by inflammatory reactions on the cellular level in the hippocampus [22, 23], we suggest a pro-neurogenic effect by indomethacin treatment based on an anti-inflammatory effect on the cellular level.

According to previous studies, we observed a decreased number of new mature neurons, characterized by NeuN expression, in the DG after MPTP treatment [9, 60, 61]. We show that long-term indomethacin treatment in turn increased the survival of new mature neurons after MPTP treatment. Selective COX-2-inhibition is known to decrease the proliferation of hippocampal neuronal cells [46, 47], whereas the unselective COX-inhibition by indomethacin has been shown to restore the amount of new mature neurons after ischemia and irradiation associated with reduced microglial activation in the DG [27, 62]. We additionally demonstrate the neurogenic potential of indomethacin on hippocampal neurogenesis in a principal model for neurodegeneration. In animal models of dopamine depletion as well as inflammation alone, impaired neurogenesis correlates with a decline of hippocampus-associated cognitive performances [6–11, 63, 64]. There is also a cellular pro-inflammatory reaction in the DG following dopamine depletion [22, 23], observed as a higher number of amoeboid CD68+ cells in this study. Here, indomethacin shows an anti-inflammatory effect by reducing the number of amoeboid CD68+ cells. This is in line with previous studies, in which indomethacin treatment after irradiation or ischemia resulted in normalized numbers of activated microglia [27, 41, 44, 65, 66]. Thus, indomethacin treatment reduces the cellular inflammatory response in a model of PD thereby leading to a pro-neurogenic effect. Whether the anti-inflammatory and neurogenesis-modulating effects of indomethacin may also improve cognitive performances, needs to be tested in future studies.

As the generation of mature neurons in the DG is a multistep process, we investigated if a specific stage of neuronal development in hippocampal neurogenesis is influenced by indomethacin. The total number of newly generated cells in the DG was not changed by MPTP or indomethacin treatment in our study. This is in line with previous studies, in which neither neurotoxin nor indomethacin treatment alone alters the general amount of proliferating cells [6, 7, 9, 23, 60, 67, 68]. We observed a decreased absolute number of newly generated type 2a cells by neurotoxin treatment regardless of drug treatment but found no change in the absolute numbers of proliferating type 1 progenitor cells by MPTP or indomethacin. We assume from our previous studies that, probably due to the here selected time points of analyses, the decreased number of newly generated type 2a cells results from a preceding reduction of type 1 cells after dopamine depletion [6]. As type 2a cells are glial progenitor cells, lineage determining to the later development of new neurons in the DG [29], their reduction may contribute to an impaired neurogenesis following neurotoxin treatment despite drug treatment at later time points. Indomethacin itself seems to not influence the number of newly generated progenitor cells at these early stages of the neurogenic process. Thus, indomethacin has a pro-neurogenic effect, represented by normal neuronal differentiation and an increased amount of newly generated mature neurons after MPTP treatment.

To elucidate the underlying molecular mechanisms of an altered neurogenic process by MPTP and its restoration by indomethacin treatment, we investigated potentially involved downstream signaling pathways. We have previously shown in the experimental autoimmune encephalomyelitis murine model of the autoimmune disease multiple sclerosis as an inflammatory animal model with subsequent neurodegeneration that the reduced differentiation into mature neurons in the DG is correlated with a reduced expression of pro-neurogenic genes [69]. Based on these findings, we investigated the expression of the target genes of the Notch, Wnt, and sonic hedgehog signaling pathways as well as of neurogenic factors in the hippocampus. The results indicate that the Wnt and Notch signaling pathways, and the neurogenic bHLH transcription factor *neuroD6* are indeed involved. The expression of *lef1*, a Wnt/β-catenin effector gene, was decreased after MPTP treatment. This is in line with in vitro findings of decreased β-catenin in neuronal progenitor cells of the subventricular zone co-cultured with MPTP and microglia [70]. As a suppression of the Wnt signaling pathway results in a decreased number of newly generated type 3 cells and immature neurons [71], we suggest that downregulated Wnt signaling contributes to a reduced survival of newly generated mature neurons following MPTP treatment in the long term. Indomethacin had no significant influence on the expression of *lef1* after MPTP treatment. However, *lef1* was decreased in healthy mice by short-term indomethacin treatment. This was probably caused by a non-steroidal anti-inflammatory drugs’ (NSAID) agonizing effect of the peroxisome proliferator-activated receptor-gamma [72–74]. Previous studies observed a downregulated Wnt signaling by indomethacin in cancer cells resulting in induced apoptosis and suppressed proliferation to prevent uncontrolled tumor growth [75, 76]. Here, the downregulated *lef1* expression in indomethacin-treated healthy mice did not affect adult neurogenesis. The expression of the neurogenic gene *neuroD6*, a member of the bHLH gene family and relevant in terminal neuronal differentiation [77], promotes neuronal survival as well as the cells’ tolerance to oxidative stress by increasing mitochondrial biomass [78, 79]. Thus, indomethacin treatment regardless of neurotoxin treatment may promote the survival of newly generated neurons. In contrast to the ST group, *neuroD6* expression was downregulated by long-term indomethacin treatment regardless of neurotoxin treatment. This did not affect neuronal development within the time span observed here. *Hes5* is a primary Notch signaling pathway effector leading to impaired neurogenesis [80–82]. It is mainly expressed in putative progenitor cells type 1, 2a, and 2b, and increased Notch signaling as well as expression of the effector gene *hes5* inhibits the differentiation of these progenitor cells into mature neurons [83–86]. In the present study, the expression of *hes5* was transiently increased in neurotoxic-treated mice regardless of indomethacin treatment in the ST group. There could be consequential inhibition of further progenitor cell differentiation eventually leading to a reduced amount of mature neurons in the MPTP-treated mice in the LT group. Short-term drug administration itself reduced *hes5* expression, which may have contributed to a differentiation into mature neurons in the indomethacin-treated mice of the LT group.

Subsequently, we assessed the serum concentration of the pro-inflammatory cytokines IL-1β, IL-6, IL-17a, and TNF-α, which mainly disturb neurogenesis, as well as IFN-γ and the anti-inflammatory cytokine IL-10, which are both rather beneficial to the neurogenic process [32, 87–93]. These cytokines have been reported to be elevated in the cerebrospinal fluid as well as serum of PD patients [33, 34, 94–96]. As microglial response and T cell infiltration occur within a few days after MPTP injection [53, 96], we expected elevated cytokine levels in serum in the ST group. Instead, the serum levels of IL-17a and IL-10 were elevated in the LT group. As the majority of the pro-inflammatory and anti-neurogenic cytokines were unchanged but the anti-inflammatory and pro-neurogenic cytokine IL-10 was elevated in turn, we suggest an incipient recovery after MPTP treatment. Together with the observation by other research groups of increased cytokine concentration in the cerebrospinal fluid but not in serum after MPTP treatment [94, 97], it should be considered that there are higher cytokine concentrations altering the surrounding brain tissue after MPTP, reflected in highly expressed mRNA levels in the midbrain and striatum tissue [98–100], which is probably not detectable in C57Bl/6 mice peripherally. Here, the increased cytokine levels of IL-10 and IL-17a in the LT group may have resulted from an inflammatory response to MPTP in peripheral organs, such as the gut tissue, where macrophage infiltration and high levels of cytokines have been found for several days [101, 102].

As previously shown in the SN [103, 104], we also observed a reduction of dopaminergic neurons after neurotoxin treatment. Contrary to the DG, indomethacin treatment itself has no neuroprotective effect on dopaminergic neurons. This is in line with the investigation by Kurkowska-Jastrzebska and colleagues, where also no neuroprotective effect of indomethacin after MPTP treatment was observed [53]. In contrast, other studies have shown a prevention of dopaminergic cell loss by indomethacin or COX-2 inhibitors, when given before the MPTP treatment started [49, 51–53]. We also observed a transient pro-inflammatory reaction following MPTP, reflected by an increased total amount of microglia. Although indomethacin treatment with its anti-inflammatory effect normalized the cellular inflammatory response, it had no effect on dopaminergic cell numbers. This suggests that the neurodegeneration was caused by direct neurotoxicity of MPTP on dopaminergic cells [21]. In human studies, NSAIDs may reduce the risk of developing PD, whereas there is currently no evidence for a secondary prevention of PD [105]. Our results in the mouse model of dopamine depletion support this in part, as indomethacin given in higher total dosage or adapted intervals may also prevent secondary inflammation-mediated dopaminergic cell loss. Nevertheless, hippocampal neurogenesis was restored correlating with reduced cellular inflammation, reflected by a decreased number of amoeboid CD68+ cells, despite dopaminergic cell loss. Thus, hippocampus-related deficits after dopamine depletion may result from inflammation-mediated reduced neurogenesis rather than from altered neurotransmitter homeostasis.

## Conclusions

In summary, we demonstrated a pro-neurogenic and thereby restorative effect of indomethacin resulting in normal progenitor cell differentiation towards mature neurons despite a MPTP-induced neurotoxic and pro-inflammatory process. We suggest that the reduced level of pro-inflammation-associated cell types, the downregulated Notch signaling pathway, and the increased expression of *neuroD6* altogether contribute to the pro-neurogenic potential of indomethacin in the DG of MPTP-treated mice. Despite indomethacin treatment and its anti-inflammatory effect in the SN, there was a neurotoxin-induced dopaminergic cell loss. Regardless of that, indomethacin promoted the survival of new mature neurons in the DG. In conclusion, indomethacin might represent a therapeutic option to restore adult neurogenesis in the DG to improve hippocampus-associated deficits in neurodegenerative diseases such as PD.

## Additional files

Additional file 1: **Figure S1.** Effects of dopamine depletion and indomethacin treatment on the stages of adult hippocampal neurogenesis. Neuronal development originates from a Nestin-positive, triangular-shaped stem cell (type 1). Then, neurogenesis progresses over the stages of the putative progenitor cells (type 2a, type 2b, and type 3) and ends in the NeuN-positive mature granule cell. Neurotoxic treatment leads to a decreased number of newly generated (type 2a cells) and mature neurons, whereas indomethacin treatment afterwards promotes the development towards mature neurons. MPTP: 1-methyl-4-(2′-methylphenyl)-1,2,3,6-tetrahydropyridine hydrochloride; NeuN: neuronal nuclei. (TIF 624 kb)

Additional file 2: **Table S1.** Hippocampal gene expression analysis. Quantitative real-time PCR was performed for the effector genes *gli1*, *hes5*, and *lef1* of the sonic hedgehog, Notch, and Wnt signaling pathway, respectively, and for the neurogenic factors *neuroD6* and *ngn1*. Gene expression is displayed as fold change of mRNA levels in relation to CTR + vehicle, *n* = 5/group. A two-way ANOVA with main factors neurotoxin, drug, and their interaction was performed. A significant interaction was followed by Bonferroni post hoc test: *p ≤ 0.05, **p ≤ 0.01 compared to CTR + vehicle. CTR: control; 1-methyl-4-(2′-methylphenyl)-1,2,3,6-tetrahydropyridine hydrochloride. (DOCX 13 kb)

## Abbreviations

bHLH: Basic helix-loop-helix
BrdU: 5-Bromo-2′-deoxyuridine
COX: Cyclooxygenase
CTR: Control
DAB: 3,3′-Diaminobenzidine
DCX: Doublecortin
DG: Dentate gyrus
GFP: Green fluorescent protein
i.p.: Intraperitoneally
IFN: Interferon
IL: Interleukin
LT: Long-term treatment
MPTP: 1-Methyl-4-phenyl-1,2,3,6-tetrahydropyridine hydrochloride, 1-methyl-4-(2′-methylphenyl)-1,2,3,6-tetrahydropyridine hydrochloride
NeuN: Neuronal nuclei
NSAID: Non-steroidal anti-inflammatory drug
PBS: Phosphate-buffered saline
PD: Parkinson’s disease
PFA: Paraformaldehyde
PG: Prostaglandin
SN: Substantia nigra
ST: Short-term treatment
TH: Tyrosine hydroxylase
TNF: Tumor necrosis factor
