# Supplementary figures and images for: Indomethacin promotes survival of new neurons in the adult murine hippocampus accompanied by anti-inflammatory effects following MPTP-induced dopamine depletion
Source: J Neuroinflammation. 2018 May 26;15:162. doi: 10.1186/s12974-018-1179-4 (PMC5970532; doi:10.1186/s12974-018-1179-4)

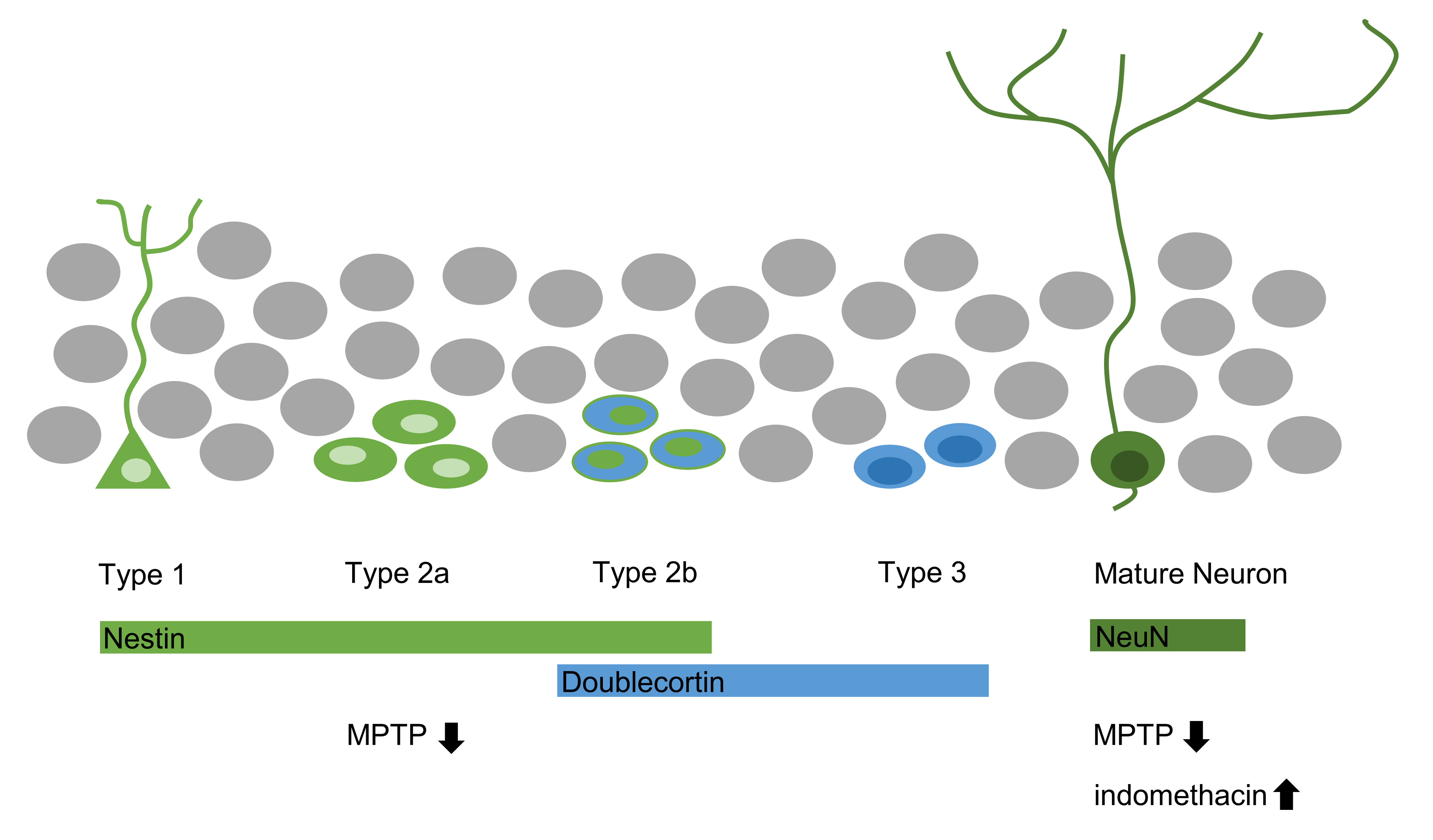

Supplement: Supplementary file 1 — Figure S1. Effects of dopamine depletion and indomethacin treatment on the stages of adult hippocampal neurogenesis. Neuronal development originates from a Nestin-positive, triangular-shaped stem cell (type 1). Then, neurogenesis progresses over the stages of the putative progenitor cells (type 2a, type 2b, and type 3) and ends in the NeuN-positive mature granule cell. Neurotoxic treatment leads to a decreased number of newly generated (type 2a cells) and mature neurons, whereas indomethacin treatment afterwards promotes the development towards mature neurons. MPTP: 1-methyl-4-(2′-methylphenyl)-1,2,3,6-tetrahydropyridine hydrochloride; NeuN: neuronal nuclei. (TIF 624 kb) [file 12974_2018_1179_MOESM1_ESM.tif]
