# Supplementary material for: Indomethacin promotes survival of new neurons in the adult murine hippocampus accompanied by anti-inflammatory effects following MPTP-induced dopamine depletion
Source: J Neuroinflammation. 2018 May 26;15:162. doi: 10.1186/s12974-018-1179-4 (PMC5970532; doi:10.1186/s12974-018-1179-4)
Supplement: Supplementary file 2 — Table S1. Hippocampal gene expression analysis. Quantitative real-time PCR was performed for the effector genes gli1, hes5, and lef1 of the sonic hedgehog, Notch, and Wnt signaling pathway, respectively, and for the neurogenic factors neuroD6 and ngn1. Gene expression is displayed as fold change of mRNA levels in relation to CTR + vehicle, n = 5/group. A two-way ANOVA with main factors neurotoxin, drug, and their interaction was performed. A significant interaction was followed by Bonferroni post hoc test: *p ≤ 0.05, **p ≤ 0.01 compared to CTR + vehicle. CTR: control; 1-methyl-4-(2′-methylphenyl)-1,2,3,6-tetrahydropyridine hydrochloride. (DOCX 13 kb) [file 12974_2018_1179_MOESM2_ESM.docx]

**Additional file 2: Table 2. Hippocampal gene expression analysis.**

|  |  | Gene | | | | |
| --- | --- | --- | --- | --- | --- | --- |
|  |  | *gli1* | *hes5* | *lef1* | *neurod6* | *ngn1* |
| Short-term treatment | CTR+vehicle | 1.0 ± 0.18 | 1.0 ± 0.14 | 1.0 ± 0.07 | 1.0 ± 0.29 | n/a |
|  | MPTP+  vehicle | 0.85 ± 0.24 | 1.47 ± 0.23 | 0.61 ± 0.12* | 1.16 ± 0.38 | n/a |
|  | CTR+  indomethacin | 0.38 ± 0.05 | 0.79 ± 0.02 | 0.49 ± 0.12** | 2.69 ± 0.82 | n/a |
|  | MPTP+  indomethacin | 0.72 ± 0.23 | 1.0 ± 0.12 | 0.64 ± 0.10 | 2.43 ± 0.17 | n/a |
| Long-term  treatment | CTR+vehicle | 1.0 ± 0.12 | 1.0 ± 0.12 | 1.0 ± 0.29 | 1.0 ± 0.14 | 1.0 ± 0.08 |
|  | MPTP+  vehicle | 0.98 ± 0.22 | 1.32 ± 0.12 | 0.71 ± 0.20 | 1.06 ± 0.15 | 1.06 ± 0.12 |
|  | CTR+  indomethacin | 0.88 ± 0.09 | 1.19 ± 0.14 | 1.02 ± 0.25 | 0.54 ± 0.03 | 0.92 ± 0.21 |
|  | MPTP+  indomethacin | 1.54 ± 0.29 | 1.12 ± 0.11 | 1.27 ± 0.16 | 0.7 ± 0.07 | 1.24 ± 0.32 |
